# Enhancement of Viscoelastic and Electrical Properties of Magnetorheological Elastomers with Nanosized Ni-Mg Cobalt-Ferrites as Fillers

**DOI:** 10.3390/ma12213531

**Published:** 2019-10-28

**Authors:** Siti Aishah Abdul Aziz, Saiful Amri Mazlan, U Ubaidillah, Muhammad Kashfi Shabdin, Nurul Azhani Yunus, Nur Azmah Nordin, Seung-Bok Choi, Rizuan Mohd Rosnan

**Affiliations:** 1 Engineering Materials and Structures (eMast) iKohza, Malaysia-Japan International Institute of Technology (MJIIT), Universiti Teknologi Malaysia, Jalan Sultan Yahya Petra, Kuala Lumpur 54100, Malaysia; aishah118@gmail.com (S.A.A.A.); nurazmah.nordin@utm.my (N.A.N.); 2 Advanced Vehicle System (AVS) Research Group, Malaysia – Japan International Institute of Technology (MJIIT), Universiti Teknologi Malaysia (UTM), Kuala Lumpur 54100, Malaysia; dekashaf@gmail.com; 3 Department of Mechanical Engineering, Faculty of Engineering, Universitas Sebelas Maret, Surakarta 57126, Indonesia; 4 National Center for Sustainable Transportation Technology (NCSTT), Bandung 40132, Indonesia; 5 Department of Mechanical Engineering, Universiti Teknologi Petronas, Persiaran UTP, Seri Iskandar 32610, Malaysia; azhani.yunus@utp.edu.my; 6 Department of Mechanical Engineering, Inha University, 253, Yonghyun-dong, Namgu, Incheon 22212, Korea; 7 Jeol (Malaysia) Sdn. Bhd., 508, Block A, Kelana Business Center 97, Jalan SS7/2, Kelana Jaya, Petaling Jaya 47301, Malaysia; rizuanmr@jeolmal.com

**Keywords:** magnetorheological elastomer, filler, hetero-aggregation, nanosized Ni-Mg cobalt ferrites, electrical resistance, resistivity, rheological properties

## Abstract

Carbon-based particles, such as graphite and graphene, have been widely used as a filler in magnetorheological elastomer (MRE) fabrication in order to obtain electrical properties of the material. However, these kinds of fillers normally require a very high concentration of particles to enhance the conductivity property. Therefore, in this study, the nanosized Ni-Mg cobalt ferrite is introduced as a filler to soften MRE and, at the same time, improve magnetic, rheological, and conductivity properties. Three types of MRE samples without and with different compositions of Mg, namely Co_0.5_Ni_0.2_Mg_0.3_Fe_2_O_4_ (A1) and Co_0.5_Ni_0.1_Mg_0.4_Fe_2_O_4_ (A2), are fabricated. The characterization related to the micrograph, magnetic, and rheological properties of the MRE samples are analyzed using scanning electron microscopy (SEM), vibrating sample magnetometer (VSM), and the rheometer. Meanwhile, the effect of the nanosized Ni-Mg cobalt ferrites on the electrical resistance property is investigated and compared with the different Mg compositions. It is shown that the storage modulus of the MRE sample with the nanosized Ni-Mg cobalt ferrites is 43% higher than that of the MRE sample without the nanomaterials. In addition, it is demonstrated that MREs with the nanosized Ni-Mg cobalt ferrites exhibit relatively low electrical resistance at the on-state as compared to the off-state condition, because MRE with a higher Mg composition shows lower electrical resistance when higher current flow occurs through the materials. This salient property of the proposed MRE can be effectively and potentially used as an actuator to control the viscoelastic property of the magnetic field or sensors to measure the strain of the flexible structures by the electrical resistance signal.

## 1. Introduction

Magnetorheological elastomer (MRE) is one of the smart materials consisting of micron size magnetic particles embedded in rubber matrix. The rheological properties of MRE can be tuned continuously and reversibly by the external magnetic field. This kind of smart material has potential applications including dampers, sensors, and actuators for vibration systems [1,2,3,4]. Currently, researchers in the field of MRE are mainly focused on the effect of different elastomer matrices [5,6,7], types of magnetic particles [8,9,10], the effect of the particle sizes [11], and the shapes of the magnetic particles [12]. Silicon rubber (SR) has been widely used in MRE fabrication as the matrix due to its softness which can lead to a high MR effect [13,14,15,16,17]. Meanwhile, carbonyl iron particles (CIPs) have been widely used in MRE fabrication due to their high magnetic saturation, high permeability, and low remanence. In addition, additives and fillers also have been introduced in MRE fabrication to strengthen the interaction between the matrix and the CIP and hence improve mechanical, rheological, and electrical properties. On the other hand, a few researchers endeavored to fabricate MRE using the nanoparticles as magnetic particles to achieve large surface area, stable chemical reaction, and easy surface modifications [18,19,20]. Mordina et al. [21] introduced two types of different shapes of CoFe_2_O_4_, spherical and fiber in silicon-based nanocomposites, and found that the nanofibers enhanced the magnetic saturation as low as 5 wt.%, but the saturation tended to decrease at a higher loading of 10 wt.% due to the low formation of interconnected network of nanofibers. Despite this, by implementing the nanoparticles as an additive or filler in MRE, the magnetic and rheological properties of MRE can be appropriately tuned. Commercially available additives such as carbon black [22,23], graphite [4,24], ester [25,26], oils [27], and carbon nanotubes [28,29,30] have been used in MRE fabrication and have proven to enhance various performances of MRE as compared to the original MRE without these additives. Aziz et al. [31] introduced a few types of multiwall carbon nanotube (MWCNTs) as an additive in MRE fabrication. The results demonstrated that with the addition of 0.1 wt.% of MWCNTs, the magnetic saturation of MRE is slightly changed, as the saturation of MRE is increased up to 4%. In a similar work, Poojary et al. [28] fabricated MRE with different percentages of carbon nanotube (CNT), 0.1 wt.% to 0.5 wt.%, as additives and found that the stiffness and loss factor was the highest with the addition of 0.25 wt.% CNT, whereas MRE without CNT exhibited the highest MR effect of 48%. Padalka et al. [32] synthesized Fe, Co, and Ni nanowires and fabricated 10 wt.% of each in MRE composite. Their study focused on the strain and frequency with compression mode showing that MRE with Fe and Co exhibited the highest MR effect at 1% strain and tended to drop at 2% strain. Meanwhile, Yu et al. [33] used the nanoparticles for MRE fabrication as a coating material for the damping applications. They synthesized CIPs which were coated with Fe nanoflakes and demonstrated that the MR effect and damping properties increased up to 162% and 65%, respectively, with the incorporation of 6% CIP-Nano-Fe.

Generally, neat MRE is considered to be an insulator, and thus electrical property can be changed by adding the proper filling material for the development of long-lasting products for sensor applications. Thus far, a few researchers have attempted to synthesize MREs associated with the nanoparticles of additives or fillers to enhance various properties such as magnetic, electrical, optical, and catalytic properties, which are known to be useful in science and technological applications [21,22,23]. For instance, cobalt ferrites nanoparticles have been extensively studied due to their variety of applications including electrical and magnetic applications. Generally, the properties of cobalt ferrite can be altered by adding dopants and metal ions, adopting a variety of synthesis methods, and varying calcination temperature and other reaction conditions. Hossain et al. [22] synthesized various Ni substituted Zn1-xNixFe_2_O_4_ by solid-state reaction technique from stoichiometric amounts of Fe_2_O_3_, ZnO, and NiO of 99.99% purity and showed that the magnetoresistance (MR) was increased with the increment of Ni-substituted Zn-Ni ferrites due to spin-dependent scattering. Berchmans et al. [23] investigated the structural and electrical properties of the magnesium-substituted nickel ferrite and found that all samples exhibited semiconductor behavior as the dielectric constantly was decreased with the frequency increment. Notably, during the last several years, the role of nanoparticles as an additive or filler has received significant research interest as sensing applications of MR-based detection methods. Nevertheless, study on this issue is considerably rare. Shabdin et al. [4] found that the application of 33 wt.% of 16 μm graphite particle size in MRE could alter the conductivity and resistivity of MRE subjected to an external applied force. Li et al. [34] fabricated MRE with different concentrations of 5.88 wt.% to 23.81 wt.% graphite and found that samples with higher graphite weight fraction showed higher electrical conductivity and lower decline in resistance. In fact, in previously published data [24,35,36], electrical properties of MRE mainly focused on the resistivity or resistance and contents of fillers. Less consideration has been given to the mechanism of particle interaction with respect to both rheological and electrical properties which are the main concern in practical applications. Although the conductivity of MRE can be improved by increasing the weight fraction of graphite, excessive implementation of particles or fillers in MRE often leads to the decrement of some other properties such as elasticity. Moreover, this makes the processing and dispersion of particles more difficult, which leads to a brittle phase and decrement of the field-dependent modulus of MRE.

The main technical contribution of this work is to fabricate a new MRE associated with the nanosized Ni-Mg cobalt ferrites with different concentrations of Mg, and to experimentally investigate various properties of the proposed MRE including the field-dependent viscoelastic properties and electrical resistance. The nanosized Ni-Mg cobalt ferrites are introduced as a filler in MRE fabrication to enhance magnetic and electric properties of MRE with the least brittleness. It should be noted that to the best of our knowledge, MRE with the nanosized Ni-Mg cobalt ferrites as a filler has never been reported, despite several benefits of the proposed filler. For example, the nanosized Ni-Mg cobalt ferrites exhibit high magnetic remanence and large surface activities [37]. Their properties are believed to demonstrate better magnetic, rheological, as well as electrical properties of MRE. In order to validate the enhanced properties of the proposed MRE, first, three MRE samples are prepared and their morphological observations are recorded. Subsequently, magnetic, field-dependent rheological, and electrical properties are investigated and compared with respect to the different compositions of Mg. It is identified that MRE with the proposed filler can enhance the saturation of magnetization by 3% and the storage modulus and loss factor by 66%. In addition, evaluations show that MRE with a higher content Mg exhibits higher electrical resistance, in the range of 1% to almost 400% in off- and on-state conditions, due to the easier movement of the particles.

## 2. Methodology

### 2.1. Samples Preparation

**Figure 1:**
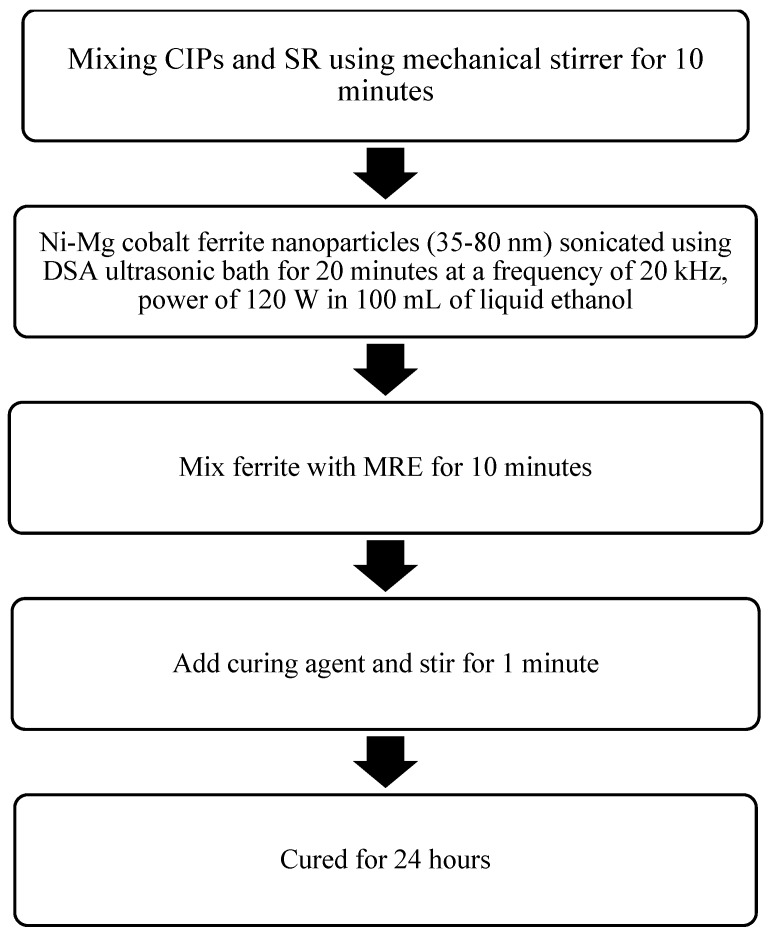
Fabrication flow chart of the proposed magnetorheological elastomer (MRE) samples.

Three types of silicone-based MRE samples were prepared through a mixing process. The CIPs with 70 wt.% of CIP were mixed with 29 wt.% of silicon rubber (SR) (NS 625 A) with the curing agent NS625B (Nippon Steel). A mechanical stirrer was used (multimix high speed dispersed (HSD), WiseStir HT-DX, PMI-Labortechnik GmbH, Lindau, Switzerland) for mixing, for approximately 10 min, until the mixture was visually seen to be homogenous. The conventional co-precipitation method was used to synthesize the nanosized Ni-Mg cobalt ferrites, namely Co_0.5_Ni_0.2_Mg_0.3_Fe_2_O_4_ (A1) and Co_0.5_Ni_0.1_Mg_0.4_Fe_2_O_4_ (A2). The procedures for the synthesized MRE samples, A1 and A2, were based on the previous method reported by Rosnan et al. [37]. The stoichiometric molar amounts of Ni(NO_3_)_2_∙6H_2_O, Mg(NO_3_)_2_∙6H_2_O, Co(CH_3_COO)_2_∙4H_2_O and Fe(NO_3_)_3_∙9H_2_O were introduced. The above-mentioned chemicals were purchased from Qrec (New Zealand) and used without further purification. The chemicals were weighted according to the required stoichiometric proportion. Then, as a filler, 1 wt.% of A1 and A2 nanosized Ni-Mg cobalt ferrites, with an average size of 35 to 80 nm, were sonicated using an ultrasonic bath (Clifton, SW6H, UK.) with a frequency of 20 kHz and power of 120 W for 20 min in 100 mL of liquid ethanol (Sigma Aldrich, Malaysia) to prevent agglomeration before mixing with the matrix and CIPs. The MRE and filler were then mixed thoroughly for another 10 min. Then, the curing agent was added, during the mixing with the SR, to the curing agent ratio, with a ratio of 95:5 and continuously stirred for another 1 min. Finally, the MRE samples were cured in the cylinder shape mold for 24 hours. The summary of the fabrication process flow is shown in Figure 1.

### 2.2. Samples Characterization

**Figure 2:**
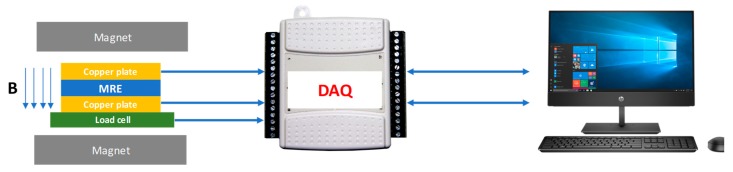
Test rig for the conductivity test.

The A1 and A2 morphological image related to the surface and structure of the nanosized Ni-Mg cobalt-ferrite particles were observed using an ultra-high resolution of Field Emission Scanning Electron Microscopy (FESEM), (JSM-7601F, JEOL, Akishima, Tokyo, Japan) at a voltage of 2.0 kV and a magnification of 50,000×. Concurrently, the cross-sections of MRE samples were observed using Scanning Electron Microscopy (SEM), (JSM-IT300LV, JEOL, Akishima, Tokyo, Japan) at a voltage of 5 kV. In order to examine the magnetic properties of all MRE samples, a Vibrating Samples Magnetometer (VSM) (MicroSense, FCM-10, LLC, MA, USA) was used to measure the magnetic properties of the MRE samples. The samples were subjected to the magnetic field in the range of −15 kA/m to +15 kA/m. Meanwhile, the effect of various magnetic fields towards the rheological properties of all MRE samples were evaluated using a rheometer (MCR 302, Anton Paar, Graz, Austria) under oscillation mode at room temperature (25 °C). All samples were subjected to different magnetic fields, ranging from 0 to 5 A (refer to Table 1) and the Tesla unit is used. The experiment is repeated for three times in order to get the consistency of the result obtained. The strain sweep test was carried out by varying the strain from 0.001% to 100%, with a constant frequency of 1 Hz, whereas another test for the current sweep was carried out by varying the magnetic fields from 0 to 5 A, with a constant strain and frequency of 0.01% and 1 Hz, respectively. The electrical resistance properties of all MRE samples (circular shape with a thickness of 1 mm, diameter of 20 mm) were evaluated using a test rig with different forces in order to analyze the electrical resistance behavior. The set-ups were almost the same as the one reported by Shabdin et al. [4]; however, a small modification has been made, in which some load cells have been attached in order to obtain the accuracy of the force, as shown in Figure 2. The test rig consists of data acquisition (DAQ) obtained from the LabVIEW software (6211, National Instruments, Austin, TX, USA). Different loads in the range of 0 to 600 g, increasing in increments of 50 g, were placed on the MRE samples in the absence and presence of a magnetic field. The test was repeated three times in order to obtain the consistency of the electrical resistance of the MRE samples.

## 3. Results and Discussion

### 3.1. Morphological Properties

**Figure 3:**
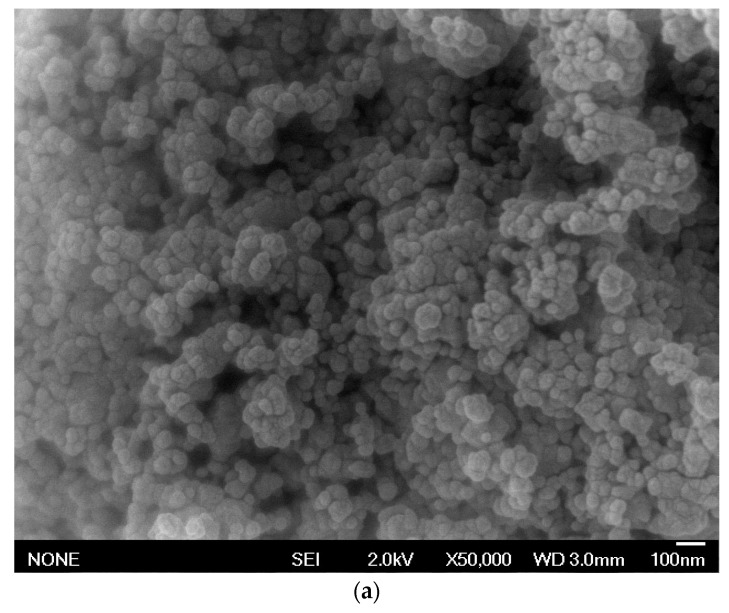

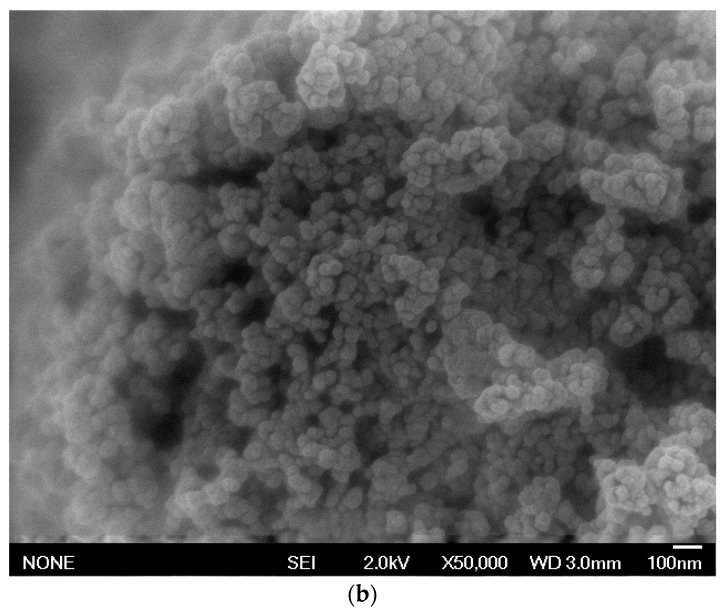
Field emission scanning electron microscopy (FESEM) images for (**a**) A1 and (**b**) A2 at 50,000× magnification at a voltage of 2.0 kV.

The morphologies related to surface and structures of Co_0.5_Ni_0.2_Mg_0.3_Fe_2_O_4_ (A1) and Co_0.5_Ni_0.1_Mg_0.4_Fe_2_O_4_ (A2) at a voltage of 2.0 kV with a magnification of 50,000× were observed in Figure 3. 

As shown in Figure 3a,b, the A1 and A2 samples exhibit spherical shapes and the nanoparticles are clearly aggregated, which is likely due to strong Van der Waals or magnetostatic interactions between bigger nanoparticles (promoted by the sintering process), a phenomenon which has been reported in previous research by Rosnan et al. [37]. In addition, by using the J-image software, the mean particle sizes observed were in the range of 35 to 80 nm. 

Meanwhile, as for the MRE with nanosized Ni-Mg cobalt ferrite, the results of the micrographic analysis for one of the samples (MRE + A2) at different magnifications (1000× and 2500× magnification) are shown below.

**Figure 4:**
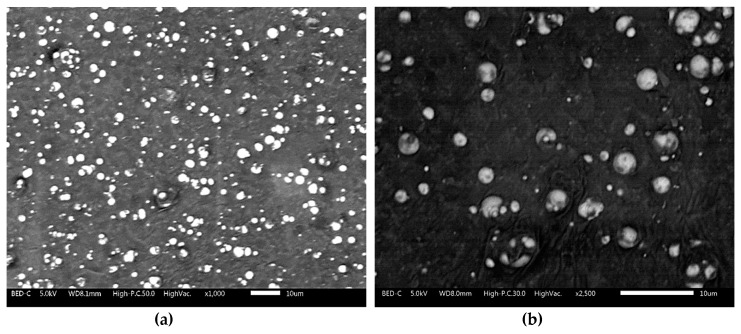
Scanning electron microscopy (SEM) images for MRE + A2 at (**a**) 1000× and (**b**) 2500× magnification.

Figure 4a displayed the dispersion of particles at 1000× magnification, while Figure 4b showed the dispersion behaviour of particles in an MRE at the higher magnification of 2500×. As demonstrated in Figure 4a,b, the CIPs and nanosized Ni-Mg cobalt-ferrites are randomly dispersed in the silicone rubber matrix. The SEM image also shows that no large aggregation or agglomeration of particles occurred while fabricating the MRE samples, although a small void is detected upon observation of the micrographic analysis.

### 3.2. Magnetic Properties

**Figure 5:**
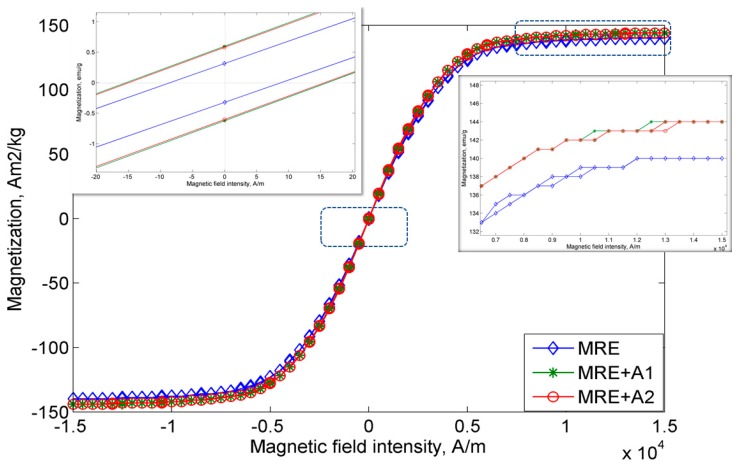
Magnetization curves for all MRE samples.

The magnetic properties of all MRE samples were measured continuously under magnetic fields in the range of −15 kA/m to 15 k/Am. Figure 5 displays the hysteresis loop of the MRE samples. As shown in Figure 5, the MRE+A1 sample exhibits the highest magnetic saturation, Ms, at 143.92 emu/g, followed by the MRE + A2 sample, at 143.77 Am^2^/kg, and control MRE at 140.25 Am^2^/kg.

The summary and comparison of all MRE samples and previous magnetic properties of A1 and A2 are shown in Table 2. The MREs with A1 and A2 exhibit an enhancement of magnetic saturation up to 2% as compared to the control MRE. Due to a higher surface area of the nanosized Ni-Mg cobalt-ferrites, it is believed that this nanoparticle fills the void between the CIPs and resulted in enhancement of the CIPs bonding. In addition, the magnetic remanence, Mr, of MRE with A1 and A2, showed a sharply decreasing value, in the range of 4200% and 4800%, as compared to the nanosized Ni-Mg cobalt-ferrite itself. As a matter of fact, lower remanence is required in any device, particularly in sensor applications as the material in the sensor is highly sensitive to the environment and must have a fast response. As such, the control MRE demonstrates the lowest remanence of 0.32 Am^2^/kg as compared to the MRE with A1 and A2. Furthermore, the coercivity, Hc, of the control MRE is also low at 8.70 Oe, while the MRE with A1 and A2 exhibit increases of 81% and 76%, respectively, in comparison to the control MRE. The increase in the coercivity and remanence of MRE with A1 and A2 are believed to be due to the high surface area of the nanosized Ni-Mg cobalt-ferrites, which resulted in easy movement of the nanosized Ni-Mg cobalt-ferrites that were attracted to the magnetic field. By implementing only these types of filler in MRE fabrication, notably, the magnetic properties exhibit larger changes, particularly in terms of remanence and coercivity behavior, which make this type of filler a potential candidate for use in the development of sensors. 

### 3.3. Sweep Strain

**Figure 6:**
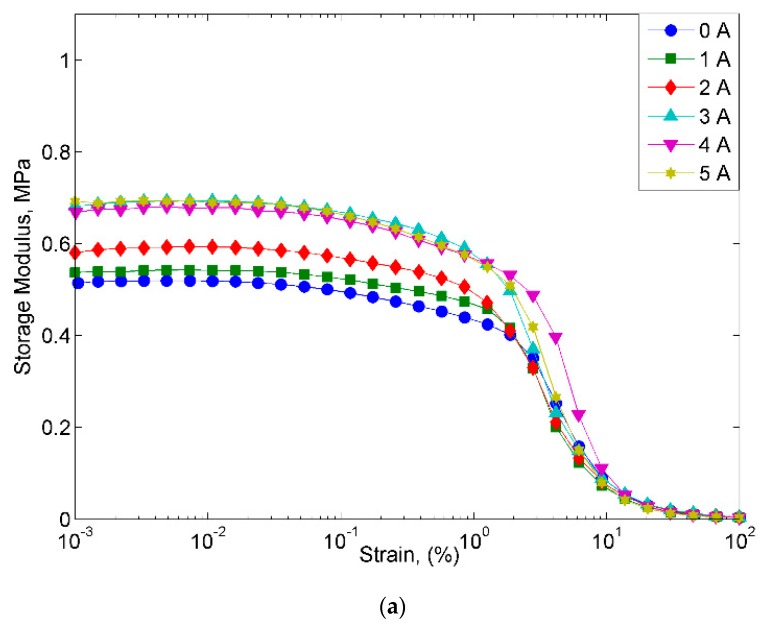

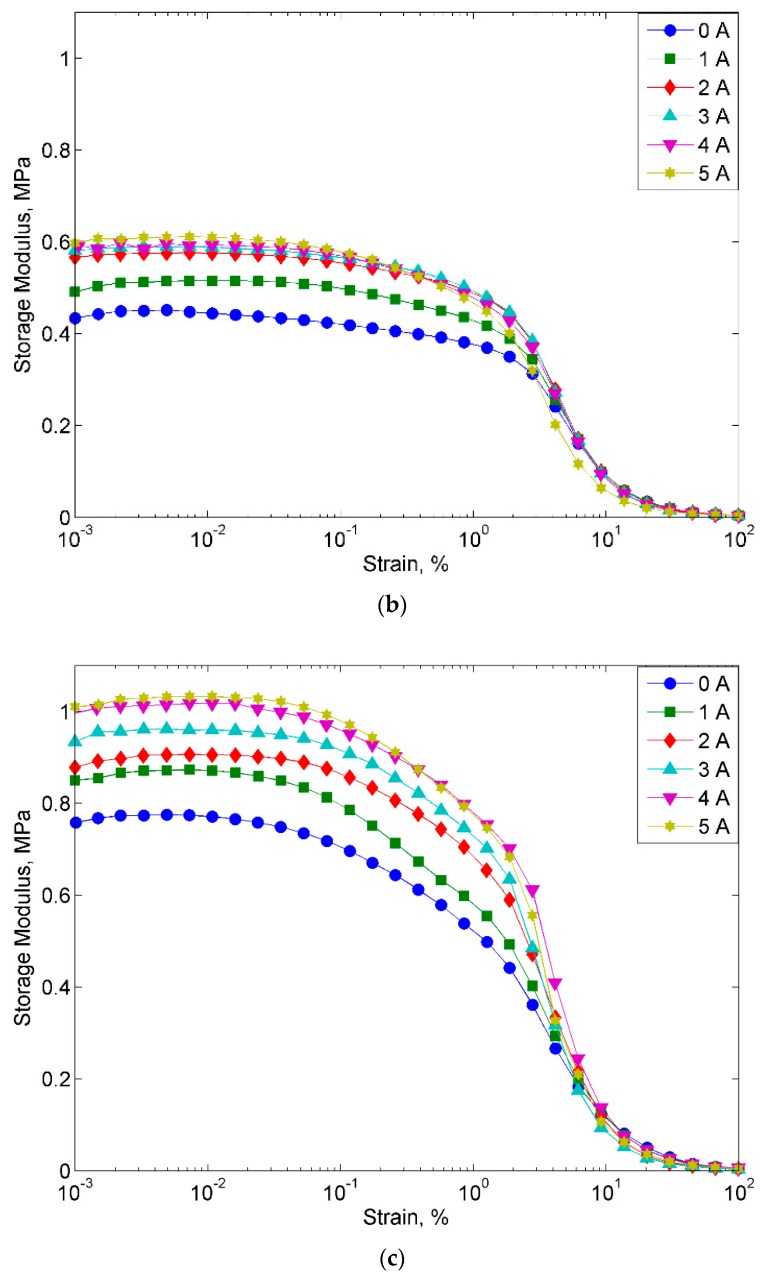
Storage modulus versus strain of all MRE samples (**a**) MRE, (**b**) MRE + A1, and (**c**) MRE + A2.

**Figure 7:**
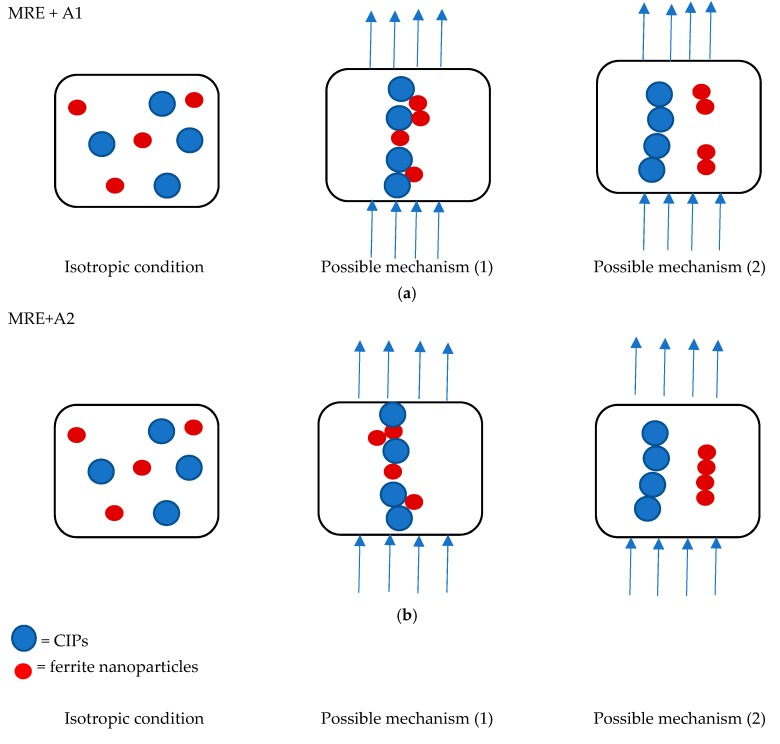
Mechanism of particle movement in the MRE samples (**a**) MRE + A1 and (**b**) MRE + A2.

Figure 6 displays the behavior of all MRE samples at different magnetic fields with the increase in the strain sweep. The MRE with A2 exhibited the highest storage modulus at all the magnetic fields applied in the off- and on-state conditions. Meanwhile, the control MRE exhibits a low storage modulus, where the decrease was up to 50% in the off-state condition and in the range of 47–50% in the on-state condition. On the other hand, the MRE with A1 demonstrated the lowest storage modulus, with 0.42 MPa in the off-state condition and a maximum storage modulus of 0.60 MPa in the on-state condition. However, the MREs with A1 and A2 follow the filler rule, in which the filler could contribute to a higher modulus of elasticity and strength than the matrix itself. Thus, in this study, the properties of the storage modulus were found to be changed by changing the weight percent of filler. A higher concentration of Mg in the nanosized Ni-Mg cobalt-ferrites increased the rheological performance of the storage modulus. However, this trend is contradicted for the MRE with A1 as the storage modulus was low as compared to the control MRE. The decrease in the storage modulus in the MRE with A1 might be due to the Ni concentration used in this MRE. As a matter of fact, the magnetic saturation of different ferromagnetic materials, namely Fe, Co and Ni has decreased, which has been reported by previous researchers [38]. It is believed that although the Ni-Mg cobalt-ferrite is able to act as reinforcing filler owing to its large surface area and high aspect ratio, the size of the nanoparticles in the MRE might have an effect on the crosslink density, thus decreasing the storage modulus. Moreover, the decreasing trend of the storage modulus of MRE + A1 might also be due to the broken filler network, which lowers the storage modulus as compared to the control sample. This finding is similar to that observed by previous researchers [39,40]. Meanwhile, for the MRE + A2, it is believed that with the addition of these nanosized Ni-Mg cobalt-ferrites in the MRE, especially in the presence of magnetic field, the hetero-aggregation process occurred between nanosized Ni-Mg cobalt-ferrites and CIPs, thus leading to an enhancement of inter-particle interactions in the MRE. In addition, the void between the CIPs may be filled by the higher surface area and smaller size offered by the nanosized Ni-Mg cobalt-ferrites, therefore, enhancing the interaction between CIPs and nanosized Ni-Mg cobalt-ferrites. A possible mechanism of the above-mentioned phenomenon is shown in Figure 7.

The mechanism of particle movement is illustrated in Figure 7. For the MRE + A1 and MRE + A2 samples, in the absence of a magnetic field, the CIPs and the nanosized Ni-Mg cobalt-ferrites tend to have a homogenous dispersion. However, when the magnetic field was applied, there are two possible situations that may have occurred. In the first—due to high magnetic saturation of the MREs—the CIPs tend to align according to the magnetic field direction, which is followed by the movement of the nanosized Ni-Mg cobalt-ferrites that will fill the void of the CIPs. In the second possible mechanism—due to the low magnetic moment of the nanosized Ni-Mg cobalt-ferrites and low Mg concentration—they will vibrate and experience slow microscopic movement which shown in Figure 7a. In this situation, the magnetic response as a result of the magnetic field will decrease and produces an obstacle in the matrix, thus reducing the storage modulus capability. Meanwhile, in the MRE + A2 sample, in the presence of a magnetic field, the nanosized Ni-Mg cobalt-ferrites will fill the void between CIPs and strengthen the interaction within the MRE. Moreover, due to high concentration of Mg, this A2 experienced faster microscopic movement which resulted in small void occurred. In this situation, due to the high Mg concentration in the nanosized Ni-Mg cobalt-ferrites, the movement of the nanosized Ni-Mg cobalt-ferrites is increased and the reaction towards the magnetic field is also increased as depicted in Figure 7b. The higher Mg content is assumed to form better bonding due to the increment of compact structure in MRE thus resulted in higher storage modulus. This kind of similar finding has been reported previously by Agarwal et al. [41] in which higher concentration resulted in better mechanical performance.

**Figure 8:**
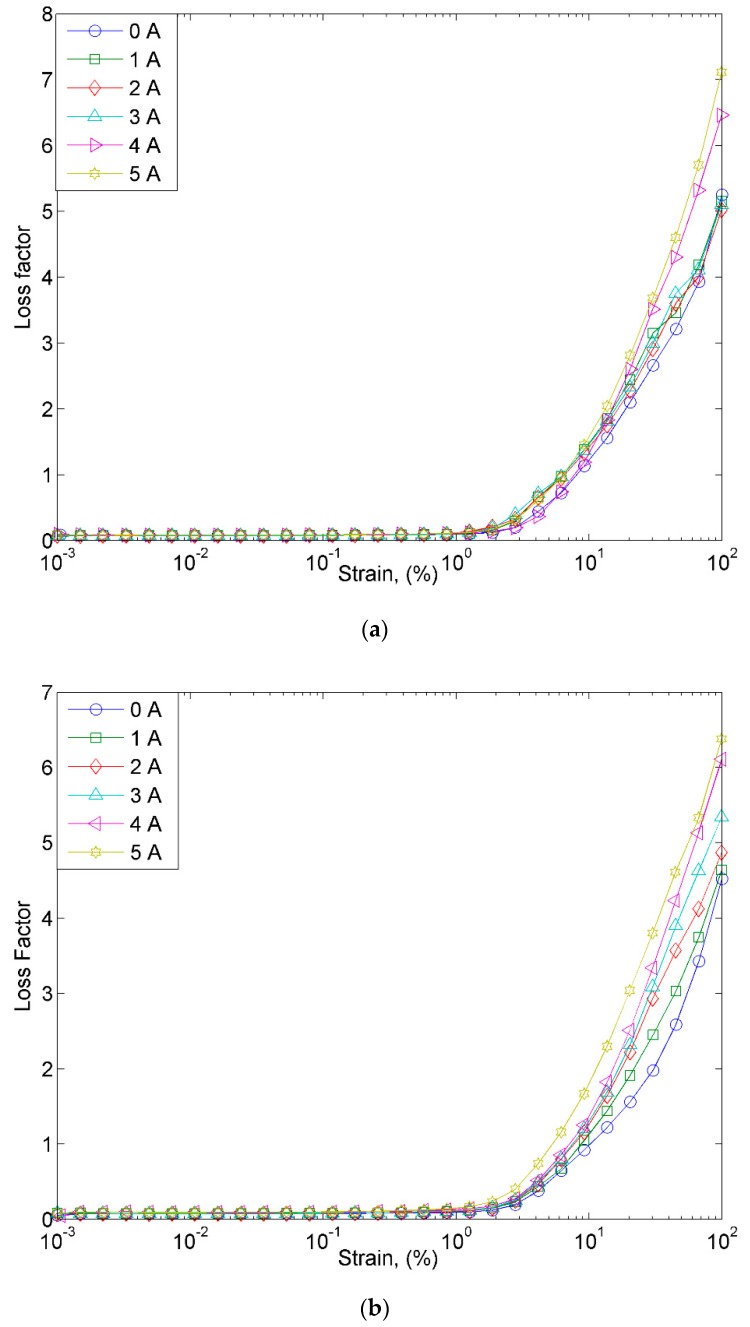

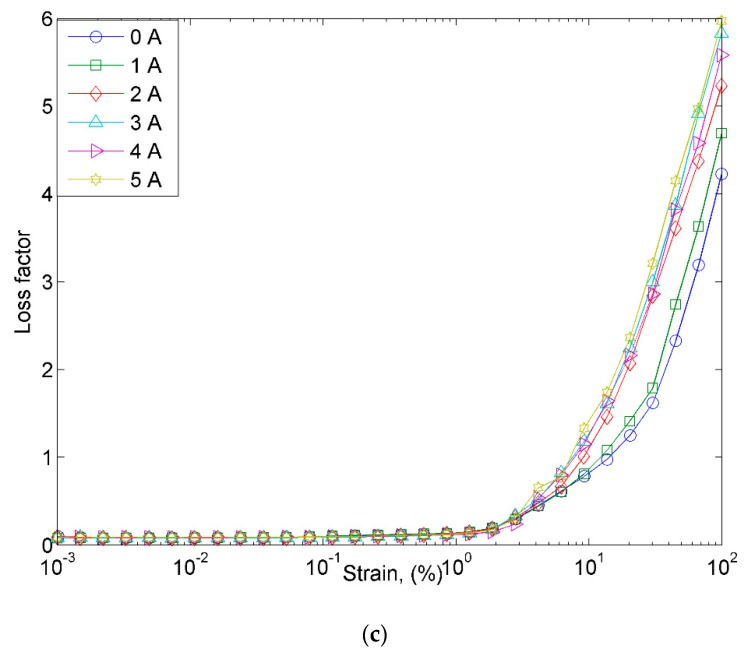
Loss factor versus strain at various magnetic fields for (**a**) MRE, (**b**) MRE + A1, and (**c**) MRE + A2.

On the other hand, the loss factor of MRE samples is shown in Figure 8. The samples were subjected to the different magnetic fields, ranging from 0 to 5 A. As shown in Figure 8a–c, it has been identified that the loss factor of all MRE samples is high at a strain of over 1%. In the off-state condition, MRE samples exhibit the lowest loss factor with the increase in the strain as compared to the on-state condition. On the contrary, in the on-state condition, MRE samples depicted a higher loss factor parallel to the increase of the magnetic field. However, the maximum loss factor decreased with the existence of nanosized Ni-Mg cobalt-ferrites in the MRE. This can be attributed to the existence of a magnetic field in which the CIPs and nanosized Ni-Mg cobalt-ferrites tended to vibrate and form a chain-like structure inside the matrix. The higher degree of macroscopic movement of the CIPs resulted in greater particle interaction and internal friction during the vibration. Thus, the energy generated from the vibrating surface of the particles was converted into heat and led to a decrease in the loss factor. Moreover, with the implementation of the nanosized Ni-Mg cobalt-ferrites in the MRE, it is believed that at a higher strain amplitude, the CIPs and nanoparticles are no longer stable due to the breakdown of the filler network. In other words, nanosized Ni-Mg cobalt-ferrites contributed to the increase in the loss factor with the increase in the strain. Therefore, it is important to determine the strain range when applying the MRE samples to the sensors, such as the strain sensor. Moreover, the response time of the sensor needs to be carefully considered if the signal from the MRE is used as a feedback signal in the control system.

### 3.4. Sweep Current

**Figure 9:**
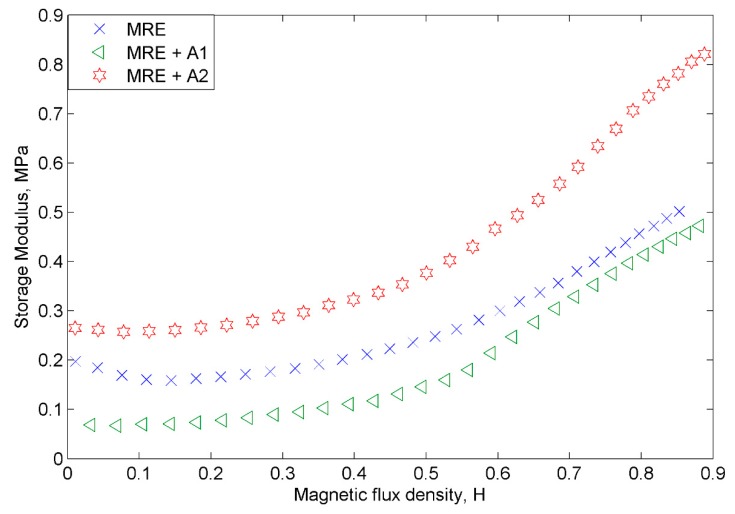
The storage modulus of all MRE samples as a function of magnetic flux density.

Figure 9 presents the storage modulus of the MRE samples at various magnetic flux densities. All MRE samples showed an increasing trend in the storage modulus parallel with the increase of the magnetic field. The summary of the rheological properties of the samples is tabulated in Table 3. The initial storage modulus of MRE + A2 showed a higher value of 0.26 MPa than the control MRE, which was at 0.19 MPa, while, the MRE + A1 exhibited the lowest initial storage modulus of 0.07 MPa. Furthermore, the MRE + A2 depicted the highest storage modulus of 0.82 MPa, followed by the control MRE at 0.50 MPa and MRE + A1 at 0.47 MPa. Both of the MRE samples with A1 and A2 show a higher MR effect—264% and 40% higher, respectively—than the control MRE. It is well known that the enhancement of the rheological properties of MRE depended on the concentration of the magnetic particles, interaction between matrix–filler and also, that of filler–filler. Therefore, the finding in this study revealed that both MREs with nanosized Ni-Mg cobalt-ferrites indicated good compatibility, interaction and bonding between matrix–filler. Due to good adhesion and binding forces between matrix-CIPs and nanosized Ni-Mg cobalt-ferrites, the macromolecular chains are restricted, thus enhancing the storage modulus, which resulted in a higher MR effect. Moreover, the general behavior of the nanosized particles—in which they tend to orient and respond faster than the larger particles—subjected to the magnetic field is also contributed to the increase in the storage modulus and magnetic effect. 

### 3.5. Electrical Resistance of the MRE Samples

**Figure 10:**
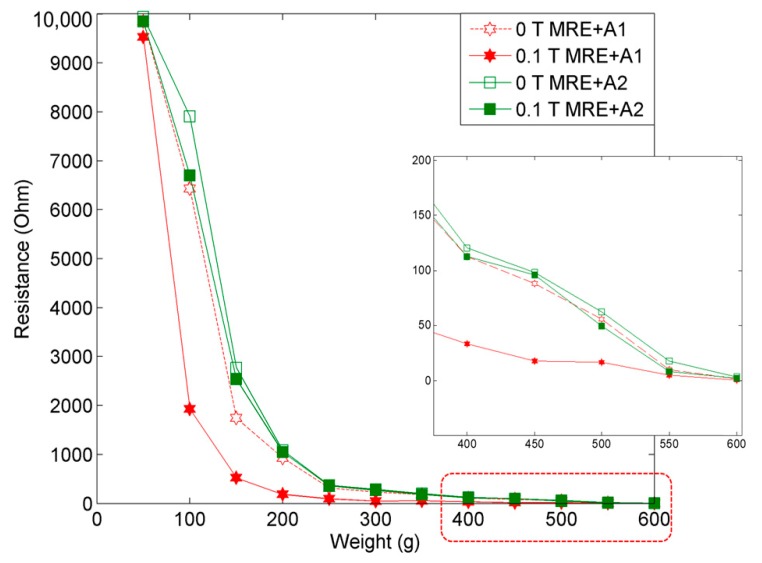
Electrical resistance comparison of MRE samples at the off- (0 T) and the on-state (0.1 T) conditions.

The electrical resistance of the MRE samples provides valuable information about the behavior of electric charge carriers, which leads to a good understanding and explanation of the conduction mechanism in MRE with nanosized Ni-Mg cobalt-ferrites. Figure 10 displays the behaviors of MRE with A1 and A2, correlated to different applied weights in the off- and on-state conditions.

The results obtained by changing the applied weight in the range of 50 to 600 g showed that the electrical resistance decreased at a higher applied weight, which proved that the MRE with nanosized Ni-Mg cobalt-ferrites is capable of exhibiting a significant change towards the electrical resistance of the MRE. Initially, in the off- and on-state conditions—(0 T) and (0.1 T)—the electrical resistances of MRE + A1 and MRE + A2 in the applied weight range from 50 to 600 g at increments of 50 g, showed an exponential decay. The summary of the electrical resistance of both MRE samples is tabulated in Table 4. 

The results demonstrated that for both the off- and on-state conditions, MRE + A1 exhibited a low electrical resistance as compared to MRE + A2, which implied that MRE + A1 is more sensitive towards the changes in force. In general, in the presence of a magnetic field, the forces between the CIPs and nanosized Ni-Mg cobalt-ferrites increased as the particles tended to form a chain-like structure. In the meantime, due to the existence of the nanosized Ni-Mg cobalt-ferrites in the MRE, the strength of the materials increased. Therefore, the mobility of these particles was restricted and led to a reduction in the electrical resistance. 

**Figure 11:**
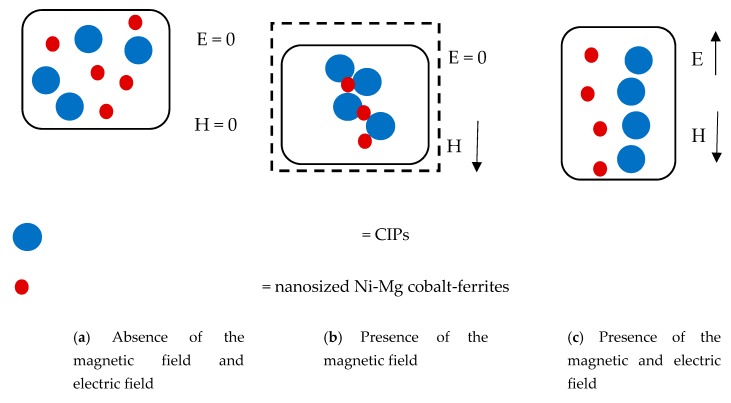
Mechanism of particles interaction in MRE with the absence and presence of magnetic and electric fields.

The mechanism of the particle interactions in the MRE samples in the absence and presence of magnetic and electric fields are shown in Figure 10. As shown in Figure 11a, during the absence of external magnetic and electric fields, the CIPs and the nanosized Ni-Mg cobalt-ferrites tend to disperse randomly in the MRE samples. However, in the presence of a magnetic field, the CIPs tend to form chain-like structures, which can be seen in Figure 11b. In addition, the nanosized Ni-Mg cobalt-ferrites fill the void between the CIPs, which enhanced the attraction force of these particles. Notably, in the presence of magnetic and electric fields (Figure 11c), the CIPs and nanosized Ni-Mg cobalt-ferrites tend to vibrate and form a chain-like structure according to the strength of the magnetic and electrical field applied. Due to the small size of the nanosized Ni-Mg cobalt-ferrites that formed in the magnetic field, the reaction is easier as compared to the CIPs. Hitherto, the CIPs and nanosized Ni-Mg cobalt-ferrites exhibit an increased magnetic moment and lead to an enhancement in the interparticle attraction force. The enhancement of this interparticle attraction force resulted in a decrease in the electrical resistance of the MRE samples, which was proven experimentally and is shown in Figure 10.

## 4. Conclusions

In this study, MRE samples with and without nanoparticles were successfully fabricated, and the overall properties, related to the magnetic, rheological and electrical resistance properties, were experimentally investigated. It has been shown from the micrographic analysis that the CIPs and nanosized Ni-Mg cobalt-ferrites are randomly dispersed in the MRE samples. More specific results achieved from this work are summarized as follows.

(1) The MRE with nanoparticles as a filler possesses an enhancement of 3% in the saturation of magnetization and decreased the remanence of the nanosized Ni-Mg cobalt-ferrite itself by 4000%. 

(2) The storage modulus and loss factor of the MRE with the nanosized Ni-Mg cobalt-ferrites showed an enhancement of almost 66% as compared to the control MRE. The results revealed that the addition of 1.0 wt.% of nanosized Ni-Mg cobalt-ferrites as filler altered and enhanced the interaction of particles in the MRE, and thus led to an increase in the magnetic and rheological properties.

(3) The electrical resistance of the nanosized Ni-Mg cobalt-ferrites decreased with the increase in the applied weight. The MRE with a higher content of Mg in nanosized Ni-Mg cobalt-ferrites exhibited higher electrical resistance in the range of 1% to almost 400% in the off- and on-state conditions due to the easier movement of the particles as a result from the deformation that occurred in the MRE matrix when exposed to an external magnetic field. 

It is interesting to note that this work demonstrates that the use of a small concentration (1.0 wt.%) of nanoparticles—which acted as a filler—is capable of altering the magnetic, rheological and electrical resistance behavior of the MRE. This capability directly indicates that MREs with appropriate nanoparticles can be used as actuators or sensors. For example, the stiffness of the flexible structure can be tuned by the MRE (actuator), and the strain (or force) of the flexible structures due to the external force can be measured using the electrical signal generated from the MRE (sensor). Future work will include the investigation of the optimum performance of the electrical resistance of larger Mg concentration and various weights of the nanosized Ni-Mg cobalt-ferrites for MRE samples. In addition, research in terms of the practical aspect of the proposed MREs with the addition of nanoparticles for actuator and/or sensor applications will be undertaken.

## Figures and Tables

**Table 1:** Magnetic flux density of the MRE samples.

Samples	Magnetic Flux Densities (T)					
	Current (A)					
	0	1	2	3	4	5
MRE	0	0.211	0.412	0.599	0.748	0.854
MRE + A1	0	0.209	0.411	0.598	0.773	0.884
MRE + A2	0	0.214	0.416	0.608	0.774	0.889

**Table 2:** Summary of the nanosized Ni-Mg cobalt ferrites and all MRE samples.

Samples	MRE	A1 [37]	MRE + A1	A2 [37]	MRE + A2
Ms (Am^2^/kg)	140.25	47.34	143.92	54.62	143.77
Mr (Am^2^/kg)	0.32	26.41	0.61	29.13	0.59
Hc (Oe)	8.70	621.27	15.71	608.17	15.33

**Table 3:** The initial modulus, absolute (Δ), and relative magnetoresistance (MR) effect.

Samples	G_0_ (MPa)	ΔG (MPa)	MR Effect (%)
MRE	0.19	0.30	157.89
MRE + A1	0.07	0.40	571.43
MRE + A2	0.26	0.56	215.38

**Table 4:** The electrical resistance of MRE + A1 and MRE + A2 with an increment of weight at the off-state (0 T) and the on-state (0.1 T) conditions.

Samples/Weight (g)	MRE + A1		MRE + A2	
	0 T	0.1 T	0 T	0.1 T
50	9848.63 Ω	9533.67 Ω	9938.91 Ω	9845.05 Ω
100	6431.33 Ω	1929.30 Ω	7906.90 Ω	6698.81 Ω
150	1744.36 Ω	523.21 Ω	2771.35 Ω	2537.91 Ω
200	929.08 Ω	185.84 Ω	1085.38 Ω	1048.48 Ω
250	314.55 Ω	94.25 Ω	369.11 Ω	359.45 Ω
300	232.83 Ω	46.43 Ω	285.56 Ω	268.98 Ω
350	180.45 Ω	54.03 Ω	200.62 Ω	183.40 Ω
400	112.82 Ω	33.67 Ω	120.34 Ω	112.86 Ω
450	88.15 Ω	17.69 Ω	98.26 Ω	96.01 Ω
500	55.68 Ω	16.58 Ω	62.66 Ω	49.30 Ω
550	10.12 Ω	5.06 Ω	17.81 Ω	8.17 Ω
600	1.73 Ω	0.20 Ω	3.02 Ω	2.37 Ω
